# Mitonuclear Communication in Stem Cell Function

**DOI:** 10.1111/cpr.13796

**Published:** 2024-12-26

**Authors:** Baozhou Peng, Yaning Wang, Hongbo Zhang

**Affiliations:** ^1^ Advanced Medical Technology Center, The First Affiliated Hospital, Zhongshan School of Medicine Sun Yat‐sen University Guangzhou China; ^2^ Key Laboratory for Stem Cells and Tissue Engineering, Ministry of Education, Zhongshan School of Medicine Sun Yat‐sen University Guangzhou China; ^3^ The Department of Histology and Embryology, Zhongshan School of Medicine Sun Yat‐sen University Guangzhou China

**Keywords:** aging, fate determination, metabolism, mitochondria, mitochondrial stress, mitonuclear communication, stem cell

## Abstract

Mitochondria perform multiple functions within the cell, including the production of ATP and a great deal of metabolic intermediates, while also contributing to the cellular stress response. The majority of mitochondrial proteins are encoded by nuclear genomes, highlighting the importance of mitonuclear communication for sustaining mitochondrial homeostasis and functional. As a crucial part of the intracellular signalling network, mitochondria can impact stem cell fate determinations. Considering the essential function of stem cells in tissue maintenance, regeneration and aging, it is important to understand how mitochondria influence stem cell fate. This review explores the significant roles of mitonuclear communication and mitochondrial proteostasis, highlighting their influence on stem cells. We also examine how mitonuclear interactions contribute to cellular homeostasis, stem cell therapies, and the potential for extending lifespan.

## Graphical abstract

In this review, we highlight the critical functions of mitonuclear communication and its impact on mitochondrial proteostasis. Additionally, we examine how mitonuclear signalling influences stem cell function, offering new insights into cellular homeostasis, stem cell therapies, and the potential for lifespan extension.

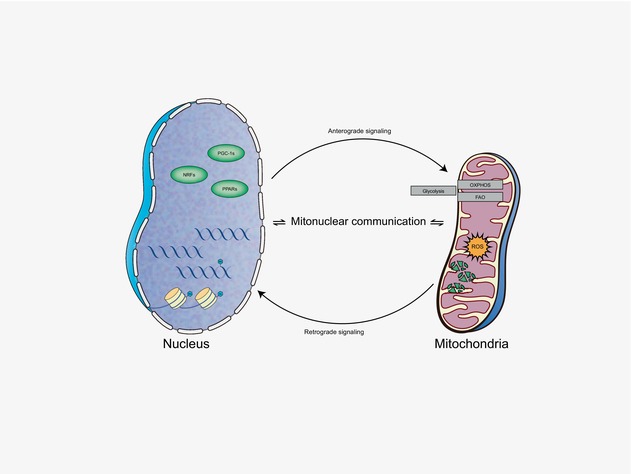

## 1 Introduction

Originated from α‐proteobacterial, mitochondria are engulfed by pre‐eukaryotic cells, and then evolve into the endosymbiont over 1 billion years ago [1]. These membrane‐bound organelles retain several bacterial characteristics of their ancestor, including an outer membrane and an inner membrane separating the intermembrane space from the matrix, small circular genomes and the ability to generate ATP via oxidative phosphorylation (OXPHOS). Over the course of evolution, several mitochondrial genes have migrated to the nuclear genome, while mitochondria acquire new components and functions from host cells. This has led to significant alterations in both mitochondria and the nuclear genome, as well as in their respective proteomes [2]. In mammals, of the more than 1000 proteins present in mitochondria, merely 13 are encoded by mitochondrial DNA. These 13 proteins are essential for all electron transport chain (ETC) complexes, except for complex II [3, 4]. The remainder of the mitochondrial proteins are encoded by genes in the nucleus, produced in the cytosol, and then directed to the mitochondria for import. Therefore, the functions of mitochondrial rely on the consistent coordination of expression of nuclear and mitochondrial genomes as well as transport and import. This coregulation is particularly crucial for the assembly of OXPHOS complexes, which consist of protein subunits derived from both genomes and require precise stoichiometric assembly [5].

Mitochondrial function is modulated by the nucleus via a mechanism known as ‘anterograde signalling’, which refers to signalling that occurs from the nucleus to the mitochondria. This signalling enhances and modulates mitochondrial activity and biogenesis to align with the requirements of cell. Conversely, mitochondria can influence nuclear gene expression via a process known as ‘retrograde signalling’, which involves communication from mitochondria to nucleus, thus allowing them to alert the nucleus to the beginning of stress to activate the proper nuclear transcriptional response. This reciprocal regulation, referred to as mitonuclear communication, forms a comprehensive network that is important for sustaining cellular homeostasis and adapting to various stressors [5, 6]. It is essential for the modulation of important processes, such as metabolism, proliferation, differentiation and the adaptation to stress.

Stem cells are special cell types that remain from embryonic stages through adulthood and into later life. They possess the remarkable capability to both self‐renew and produce specialised cell types in vivo via asymmetric cell division [7, 8]. Under normal conditions, most stem cells exist in a quiescence state, characterised by reduced metabolic activity and temporary cell‐cycle arrest. The processes of exiting and re‐entering quiescence are tightly regulated by both external signals and internal quality control mechanisms, which can be disrupted by aging and various diseases [9]. It is generally accepted that metabolism is thought to be accompanied by alternations in stem cell fate to meet the distinct metabolic requirements of stem, progenitor or differentiated cells [10]. Nevertheless, researches over recent decades have demonstrated that changes in metabolism can significantly affect stem cell fate and functional, akin to the role of transcriptional networks in determining stem cell characteristics [11]. Meanwhile, as time passes, stem cells gradually diminish in their plasticity due to replicative and chronological senescence, which impacts tissue function. This decline can manifest as slower wound healing, reduced muscle strength, weakened immune response, and greying or loss of hair [12, 13]. Notably, the 12 widely accepted hallmarks of aging identified by scientists encompass mitochondrial dysfunction and stem cell exhaustion, both of which are essential factors in the changes associated with aging [14, 15].

Over the past decades, interest in the intricate communication between mitochondria and nucleus has grown significantly due to their critical involvement in health and lifespan [6]. Nevertheless, ongoing research still aims to unravel the complex relationships between mitonuclear communication and metabolic changes of stem cells. In this review, we explore the powerful functions of mitonuclear communication including anterograde signalling and retrograde signalling. Moreover, we focus on the effects of mitochondrial proteostasis. Furthermore, we discuss new advances in linking mitonuclear communication to stem cell function, including energy metabolism, mitochondrial dynamics, epigenetics and proteostasis. These advances will expand our understanding of mitochondria in stem cells and aid in advancing regenerative medicine and addressing age‐related diseases.

## 2 Anterograde Signalling

**FIGURE 1:**
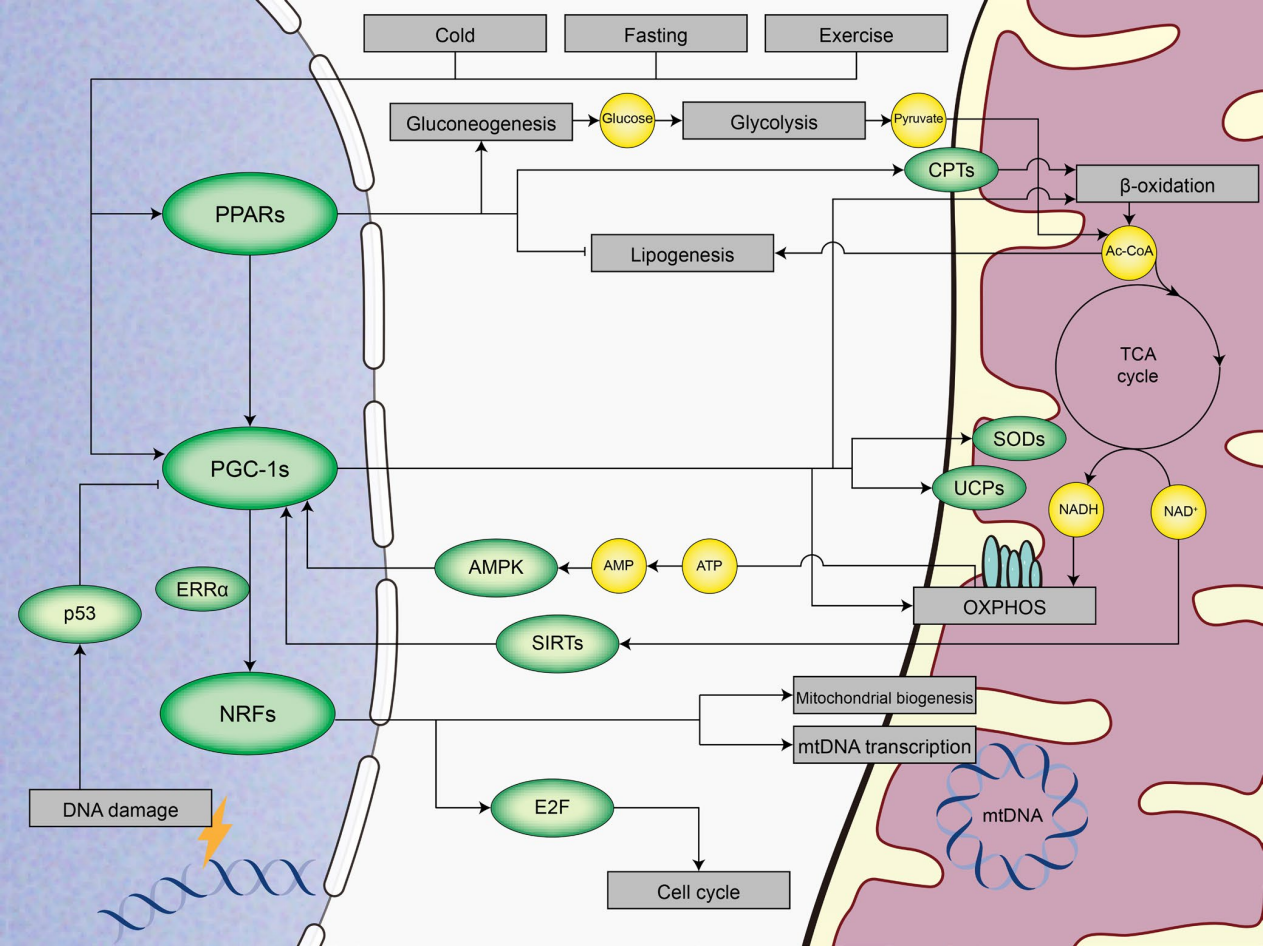
General view of anterograde signalling. Anterograde signalling regulates mitochondrial function to adapt to the cellular environment. PPARs serve as key regulators of fatty acid metabolism, activating fatty acid catabolism and stimulating gluconeogenesis. PGC‐1 proteins are crucial for mitochondrial metabolism, as they are key to regulate the expression of various enzymes that detoxify ROS. Meanwhile, it can also modulate mitochondrial biogenesis via oestrogen‐related receptor alpha (ERRα) and NRFs. In addition, NRFs target genes that encode elements of the mtDNA transcription machinery, including OXPHOS complexes and protein import system.

The function of mitochondrial is regulated by the nucleus via anterograde signalling, allowing these organelles to respond to the cellular environment (Figure 1). The regulatory mechanism mainly depends on the expression of mitochondrial proteins encoded by nuclear DNA. These proteins subsequently stimulate the transcription of mtDNA genes. Various transcription factors and coregulators are also vital for managing mitochondrial proteome. Key regulators of these nuclear‐encoded mitochondrial proteins include NRFs, PPARs, and PGC‐1 family members.

### 2.1 Nuclear Respiration Factors (NRFs)

The expression of mitochondrial proteins encoded by nuclear DNA is primarily controlled by two transcription factors: nuclear respiration factors 1 (NRF1) and nuclear respiration factor 2 (NRF2, also known as GABP).

#### 2.1.1 NRF1

In the early 1980s, NRF1 was identified as a positive transcriptional regulator while studying the cytochrome c promoter [16, 17]. As a homodimer, NRF1 possesses a distinctive DNA‐binding domain along with an essential carboxy‐terminal transcriptional activation domain enriched with glutamine and hydrophobic amino acid clusters [18]. NRF1 is involved in regulating numerous genes essential for mitochondrial respiratory activity, including those coding five subunits of the respiratory complex, and others that facilitate the expression, assembly, and function of the respiratory chain [19]. Of note, NRF1 influences human genes related to the mitochondrial DNA transcription machinery, such as *TFAM*, *TFB1M*, *TFB2M*, and *POLRMT* [20]. Furthermore, it is linked to the expression of enzymes in the heme biosynthesis pathway and elements necessary for protein assembly and import [21]. These genes have potential integrative functions in the synchronised expression of both mitochondrial and nuclear respiratory components, supporting the idea that NRF1 is pivotal in mitonuclear interactions [22]. Physiological studies have further demonstrated NRF1 critical involvement in regulating mitochondrial biogenesis and function, as evidenced by targeted deletion of the NRF1 gene in mice leading to early embryonic death and significant loss of mtDNA [22, 23].

A genome‐wide analysis has identified that a number of human promoters are in vivo occupied by NRF1, with many of these genes playing roles in mitochondrial metabolism and biogenesis [24]. Interestingly, a notable group of the NRF1 target genes are associated with E2F, a growth regulatory transcription factor, indicating NRF1 involvement in the modulation of certain E2F responsive genes. These genes are necessary for cell cycle, and NRF1 siRNAs have been shown to repress the expression of some of these E2F targets [24]. Of note, NRF1 gets phosphorylated when cell enters the cell cycle, while remain dephosphorylated in quiescent state. And this phosphorylation, occurring at several serine residues within a specific amino‐terminal region, boosts NRF1 DNA‐binding and trans‐activation functions [25, 26]. This, together with the de‐repression of E2F factors released by some NRF1 target genes, may support increased cell proliferation.

Some evidences prove the function of NRF1 in stem cells and development. In foetal development, redox regulation in myoblasts is crucial for preventing reactive oxygen species (ROS)‐induced damage during differentiation, which mechanism is the direct regulation of NRF1 by PITX2/3 [27]. Equally, NRF1, mediating a dual stress response that activates the proteasome and redox balance, is indispensable for cardioprotection and neonatal heart regeneration in adult [28]. In the rat retina, NRF1 also regulates the transcription of the *CXCR4*, a gene essential for multiple physiologic and pathologic functions of eye, like immune responses, ocular neovascularisation and neuronal development [29]. This relationship between NRF1 and CXCR4 likewise promotes breast cancer stem cells (CSCs) growth [30]. Meanwhile, knockdown of NRF1 resulted in reduced expression of GABPβ and BRCA1, both of which have been identified important for mammary stem/progenitor cell differentiation [31]. Intriguingly, in haematopoietic stem cells (HSCs), SIRT7 is induced under mitochondrial protein folding stress, helping to alleviate stress by keeping NRF1 activity in check, which promote stem cells maintenance [32, 33, 34]. Besides, NRF1 is also critical in the neurodevelopmental process and the development of post‐migrating primordial germ cells [35, 36].

#### 2.1.2 NRF2

NRF2, the second nuclear respiratory factor, is known for its ability to specifically bind to crucial cis‐acting elements in the *COXIV* promoter [37]. The recognition sites for NRF2 in the *COXIV* promoter of rodent contain the core GGAA motifs, typical of the ETS domain transcription factors family [38]. Purifying NRF2 from nuclear extracts of HeLa cell leads to the identification of five subunits: α, β_1_, β_2_, γ_1_, and γ_2_ [39]. The α subunit interacts with DNA, while the others form heterodimeric and heterotetrameric complexes with it. NRF2 is identified as the human equivalent of mouse GABP, which is a transcription factor composed of three subunits (α, β_1_, β_2_) with ETS domains, with human β_2_ and γ_2_ corresponding to GABPβ_1_ and β_2_, respectively [40, 41]. The first known cellular function of NRF2 is to activate the expression of COX subunits [37]. A shRNA directed against *NRF2* reduced expression of all 10 nuclear‐encoded cytochrome oxidase subunits consist with the fact in a promoter mutation study [42, 43]. However, the mitochondrial action of NRF2 is not limited to COX expression.

NRF2 also targets numerous genes necessary for respiratory chain function, including those coding for mitochondrial transcription factors like TFAM and TFB isoforms, and three out of the four human succinate dehydrogenase subunit genes [20, 44, 45, 46, 47]. Furthermore, NRF2 involves in the expression of membrane receptor complex subunits essential for importing proteins that promote various mitochondrial functions, suggesting its broad role in coordinating mitochondrial biogenesis [48, 49, 50]. As previously observed in NRF1‐null mice, NRF2‐null mice display a lethal phenotype during peri‐implantation [51]. This suggests that NRFs transcription factors fail to compensate for each other in embryonic development. Interestingly, NRF2 also participates in a D‐cyclin‐independent pathway for cell cycle entry, linking NRF‐dependent transcriptional expression with the cell proliferation related to respiratory function [52].

In view of the important function of NRF2 in mitochondria, it also has a pivotal role in stem cells. Through downregulation of *Oct4* repressors in mouse embryonic stem cell (ESCs), GABPα upregulates *Oct4* expression which is important for maintaining pluripotency and self‐renewal [53]. Further evidence comes from the drastically repressed proliferation and cell death within 2 days in GABPα‐null embryonic stem cells [54]. Likewise, GABPα‐null mice experience a rapid decline in myeloid cells, with any remaining myeloid cells appearing dysplastic and immunophenotypically abnormal. This study highlights the crucial role of GABP in myeloid differentiation, partly, through its regulation of GFI1 and ITGAM [55, 56]. Meanwhile, a GABP‐controlled gene regulatory module has been identified, influencing various aspects of HSCs biology, such as maintenance and multipotency [57]. Consisting with it, other studies show that GABPβ_1_/β_2_ deficiency specifically impairs HSCs quiescence and survival [58, 59]. In addition, knockdown of GABPα results in an increase in GABPβ1, while reducing GABPβ1 significantly decreases the proliferation of neural stem cells (NSCs) and promotes neuronal and astrocytic differentiation, suggesting its vital role in regulating the balance between proliferation and differentiation of NSCs [60, 61].

### 2.2 Peroxisome Proliferator‐Activated Receptors (PPARs)

Peroxisome proliferator activated receptors (PPARs) are not only nuclear hormone receptors but also transcription factors activated by ligands. They are classified as type II members of the nuclear hormone receptor superfamily, which is part of the steroid/thyroid hormone receptor gene superfamily. The PPARs family includes three primary isotypes: PPARα, PPARβ/δ and PPARγ. All three isoforms share a similar protein domain structure, which is typical of many members of this superfamily. Key domains include DNA‐binding and ligand‐binding domain [62]. The ligand‐binding domain features 12 α‐helices that create a distinctive three‐layer antiparallel α‐helical structure, accompanied by a compact four‐stranded sheet [63]. This arrangement forms a sizable Y‐shaped hydrophobic pocket, known as the ligand‐binding cavity, which likely facilitates the binding of a diverse array of lipophilic compounds.

#### 2.2.1 PPARα

PPARα distributes in the tissues that exhibit significant fatty acid metabolism and peroxisomal activity including heart, liver, kidney, brown adipose tissue, skeletal muscle and intestine, where it is essential for regulating energy homeostasis [64, 65]. PPARα serves as the primary regulator of fatty acid utilisation, promoting fatty acid catabolism, enhancing ketone body production and gluconeogenesis, and also influencing lipoprotein assembly [62]. PPARα activates genes related to the transport of fatty acids such as *CPT1* and *CPT2*, along with key enzymes in the β‐oxidation pathway like *ACAD*, *HADH*, *DCI* and *DECR* [66]. Through β‐oxidation, fatty acids are converted into acetyl‐CoA (Ac‐CoA). During fasting, PPARα enhances the use of Ac‐CoA in mitochondria of liver for ketone bodies production, which peripheral tissues then utilise in the tricarboxylic acid cycle, via activating HMG‐CoA Synthase [67]. The positive effects of PPARα‐mediated mitochondrial fatty acid disposal are well‐documented in models of NAFLD/NASH [68], where increased fatty acid oxidation (FAO) leads to reduced circulating fatty acid and triglyceride levels, decreased liver and muscle fat accumulation, and improved insulin sensitivity [69, 70]. Furthermore, PPARs promote the expression of mitochondrial uncoupling proteins, enhancing energy expenditure through futile FAO [71]. It is noteworthy that fibrate drugs, commonly prescribed for hypertriglyceridemia, act as PPARα activators [62]. Furthermore, PPARα agonists exhibit significant anti‐inflammatory properties, contributing to their protective effects in the cardiovascular system [70].

There is limited knowledge about endogenous ligands for PPARα, and its specific role in stem cells remains unclear. Most strikingly, activation of PPARα in conjunction with the glucocorticoid receptor has been demonstrated to support erythroid progenitors self‐renewal and can alleviate anaemia in a mouse chronic anaemia model [72]. The 4‐phenylbutyric acid can enhance PPARα activity, initiating liver CSCs via the activation of β‐catenin signalling pathway, which promotes hepatocellular carcinoma at early stage [73, 74]. Furthermore, microbial danger signals from intestinal inflammation can be detected by the liver, leading to decreased production of PEDF by PPARα and this repression allows for increased proliferation of intestinal stem cells (ISCs), thereby accelerating tissue repair in the gut [75].

#### 2.2.2 PPARβ/δ

PPARβ/δ exhibits the most extensive expression pattern among the PPARs, with expression levels varying in different tissues based on cell proliferation and differentiation [62]. PPARβ/δ has similar functions as PPARα in other tissues, particularly in adipose and muscle tissues, stimulating the expression of enzymes related to mitochondrial FAO. In mouse models, agonists of PPARβ/δ have been shown to slow weight gain with high‐fat diet, helping to preserve insulin sensitivity by enhancing fatty acid metabolism and thermogenesis of skeletal muscle [76, 77]. Also, PPARβ/δ acts as a crucial transcription factor, which can mitigate effects of inflammatory cytokines like TNFα and IFNγ at injury sites [78].

In recent years, an increasing amount of evidences have emanated that PPARβ/δ modulators impact on stem cells, regulating development, regeneration and homeostasis. PPARβ/δ is required for the development of brain, skin, placental and gut [79, 80, 81, 82]. In adult epidermis, PPARα and β/δ expression is reactivated in response to stimuli like hair plucking or skin wounds, promoting keratinocyte proliferation and differentiation, thus revealing new roles in epidermal repair [83]. During wound healing process, PPARβ/δ protects keratinocytes from apoptosis via Akt1 activation, aiding skin wound closure [84, 85]. Located in the lower region of each small intestinal crypt, ISCs show high expression of PPARβ/δ [82]. Meanwhile, in ISCs and progenitor cells, pharmacological activation or high‐fat diet induction of PPARα and PPARβ/δ enhances the stemness and tumorigenicity [86]. Conversely, the loss of PPARβ/δ or inhibition of mitochondrial FAO impairs the maintenance of HSCs, while treatment with PPARβ/δ agonists supports HSCs maintenance and increased asymmetric cell division [87]. In mouse ESCs, PPARβ/δ is critical for mitochondrial biogenesis and differentiation into hepatic‐like tissue [88]. Its activation in osteoblast and mesenchymal stem cells (MSCs) boosts Wnt signalling activity and osteoblast differentiation while also acting as a negative regulator of MSCs immunosuppressive functions [89, 90]. Additionally, PPARβ/δ participates in muscle stem cells (MuSCs) proliferation and myoblast fusion [91]. In muscle tissue, PPARβ/δ overexpression, as well as its pharmacological activation, enhances skeletal muscle fusion without affecting MuSC proliferation [92]. This aligns with findings that PPARβ/δ activation enhances C2C12 differentiation through the AKT and p38 MAPK pathways [93]. The lack of PPARβ/δ leads to a reduction in MuSCs, impairing postnatal myogenesis and skeletal muscle regeneration [94]. Mice lacking PPARβ/δ display decreased proliferation of MuSCs, yet demonstrate enhanced differentiation. This effect is associated with the downregulation of FOXO1, which inhibits skeletal muscle differentiation [95].

#### 2.2.3 PPARγ

There are two isoforms of PPARγ: PPARγ1 and PPARγ2. PPARγ2 is predominantly found in the adipose tissues, whereas PPARγ1 has a more widespread distribution, including in the gut, vascular cells, and certain immune cells [62]. Notably, PPARγ is vital for regulating overall glucose and lipid homeostasis by its coordinating activities in the muscle adipose tissue and liver. This regulation is supported by an intricate network of interactions involving circulating lipids, inflammatory cytokines, adipokines and hepatokines [96]. PPARγ is also important for modulating the inflammatory response, particularly within dendritic cells and macrophages [97, 98, 99, 100].

Of note, PPARγ is critical for regulating cell proliferation, differentiation, and survival. PPARγ is a key player in the differentiation of adipose tissue and in sustaining particular functions of adipocyte. In mouse models with the white adipose tissue knocking‐down of PPARγ, the mice exhibit growth retardation and develop pronounced lipodystrophy and hyperlipidaemia [101]. Moreover, mature white and brown adipocytes lacking PPARγ undergo apoptosis within a few days, and are subsequently replaced by newly generated PPARγ‐positive adipocytes, highlighting its vital role in the survival of mature adipocytes in vivo [102]. And during aging, PPARγ is upregulated in protein level in mouse MuSCs, while PPARγ is the main switch in adipogenic differentiation of MSCs [103, 104]. In tune with it, PPARγ‐null ESCs are unable to differentiate into adipocytes, instead differentiating to osteoblasts [105]. Antagonism of the PPARγ pathway promotes the growth of HSCs and progenitor cells from human cord blood by boosting glycolysis and inhibiting differentiation [106]. PPARγ signalling safeguards hair follicle stem cells against apoptosis caused by chemotherapy [107]. Meanwhile, targeted removal of PPARγ in hair follicular stem cells leads to a skin and hair phenotype resembling scarring alopecia, underscoring its importance for maintaining healthy pilosebaceous units [108]. Moreover, preconditioning with the PPARγ agonist rosiglitazone helps maintain the functions of transplanted stem cells under an oxidative stress environment, enhancing organ regeneration by reducing inflammation and oxidative stress damage [109].

### 2.3 Peroxisome Proliferator Activated Receptor Co‐Activators (PGC‐1s)

Peroxisome proliferator activated receptor co‐activators (PGC‐1s), which include PGC‐1α, PGC‐1β and PGC‐1‐related coactivator (PRC), serve as essential regulators of mitochondrial function, integrity and biogenesis [110]. Each of these PGC‐1 isoforms features an N‐terminal activation domain with leucine‐rich LXXLL motifs, which facilitate interactions with nuclear receptors, as well as a binding domain for host cell factor‐1 and an RNA recognition domain. Notably, PGC‐1α also has an RNA splicing domain, although its specific role remains to be elucidated [111].

#### 2.3.1 PGC‐1α

PGC‐1α, the most extensively studied isoform, is first identified as a coactivator of PPARγ in brown adipose tissue when exposed to cold, where it is pivotal for managing adaptive thermogenesis and mitochondrial activity [112]. It can be triggered by various external stimuli that heighten energy demands, like fasting, exercise, and cold temperatures [113]. The prevailing hypothesis suggests that PGC‐1α associates with certain transcription factors within the promoter regions of target genes, recruiting complexes such as P300 and TRAP/DRIP to facilitate chromatin remodelling through histone acetylation, thereby initiating transcription via RNA polymerase II [114]. One of the most well‐defined roles of PGC‐1s is their promotion in mitochondrial biogenesis through the coactivation of several transcription factors, including oestrogen‐related receptor alpha (ERRα) and NRFs [115, 116, 117]. These transcription factors subsequently control the expression of TFAM, which is crucial for replication, maintenance and mtDNA transcription [118]. Also, PGC‐1α serves as a potent regulator of ROS metabolism, being necessary for the activation of various ROS‐detoxifying enzymes such as GPX1 and SOD2 [119]. Consisting with it, evidence suggests that signalling pathways associated with PGC‐1α are disrupted in muscular dystrophy, resulting in decreased mitochondrial oxidative phosphorylation and increased ROS production [120]. Studies on PGC‐1α overexpression in adipocytes, cardiac myocytes, and mouse hearts indicate its capacity to drive all facets of mitochondrial biogenesis, including the activation of genes related to FAO and respiratory chain, along with an increase in mitochondrial numbers and respiratory capacity [24, 121, 122]. Reportedly, PGC‐1α‐null mice struggle to maintain body temperature during cold exposure and exhibit slow‐twitch skeletal muscles and impaired growth of the heart and after birth, along with a reduced ability to sustain cardiac output and exercise performance in slow‐twitch muscle [123]. Moreover, PGC‐1α is modulated at the post‐translational level through various protein modifications, including phosphorylation, acetylation, methylation, ubiquitination and SUMOylation. For instance, phosphorylation by AMP‐activated protein kinase (AMPK) and p38 MAPK enhances its transactivation activity and reduces its degradation [124, 125]. AMPK also facilitates the activation of SIRT1, which boosts PGC‐1α transcriptional function [126, 127]. Conversely, phosphorylation by AKT can inhibit PGC‐1α activity by preventing its recruitment to target promoter regions [128]. Furthermore, acetylation by GCN5 also represses PGC‐1α function [129]. In addition, members of PGC‐1 family generally have short half‐lives due to rapid ubiquitination and proteasomal degradation [130].

Emerging evidence shows that PGC‐1α participate in stem cell function, development and homeostasis. For instance, PGC‐1α overexpression can active the anti‐apoptotic gene Bcl‐2 while inhibiting the pro‐apoptotic gene Bax by interacting with ERRα, as well as promoting the survival of MSCs [131]. In muscle‐specific PGC‐1α transgenic mice, although MuSCs number is lower, those that are present show increased activation and proliferation, indicating that PGC‐1α supports the early stages of MuSC activation [132, 133]. Another research shows that transduction of skeletal muscle with PGC‐1α has been demonstrated to enhance the formation and size of neuromuscular junctions (NMJ), primarily by increasing the secretion of neurturin, which is essential for PGC‐1α effects on NMJ formation [134]. Notably, PGC‐1α levels declines with age in skeletal stem cells of both human and mouse. The reduction of PGC‐1α results in an elevation in adipogenic differentiation while compromising osteoblastic differentiation. Conversely, boosting PGC‐1α levels can mitigate bone loss associated with osteoporosis and reduce marrow adiposity [135]. It has been reported that pharmacological activation of PGC‐1α can enhance muscle strength and oxidative capacity, contributing to metabolic rejuvenation in aged mice [136]. Moreover, pharmacological activation of PGC‐1α promotes a maturation metabolic shift from glycolysis to OXPHOS of cardiomyocytes derived from human ESCs via YAP1 and SF3B2 [137, 138, 139, 140]. Analogously, the balance between PGC‐1α and MYC is crucial for determining the adaptability and metabolic state of pancreatic CSCs, which rely more on OXPHOS than on glycolysis [141]. In zebrafish, PGC‐1α is critical for determining renal progenitor cells fate and is necessary for proper nephron segment patterning during kidney development [142]. It supports ciliogenesis in different cell types, like nodal, monociliated, and multiciliated cells, and influences renal tubule ciliated cells fate during embryonic development. Interestingly, treatment with PGE2 can restore cilia formation and multiciliated cell fate in PGC‐1α‐null zebrafish, highlighting its role in prostaglandin signalling [143]. Additionally, overexpression of the PGC‐1 homologue dPGC‐1 in *Drosophila* ISCs has been linked to increased lifespan and enhanced mitochondrial activity, which leads to improved tissue homeostasis in aged flies [144].

#### 2.3.2 PGC‐1β

PGC‐1β, also known as PGC‐1‐related oestrogen receptor coactivator (PERC), shares significant sequence similarities with PGC‐1α and is capable to regulate several of the same downstream targets [145]. Like PGC‐1α, PGC‐1β is mainly found in tissues rich in mitochondria, such as skeletal muscle, heart and brown adipose tissue, though it is not elevated in brown adipose tissue when exposed to cold [145]. Notably, PGC‐1β preferentially enhances the ligand‐dependent function of oestrogen receptor alpha (ERα), with only a minor impact on ERβ [146]. Mice lacking PGC‐1β display mitochondrial dysfunction under stress, with a marked reduction in nuclear‐encoded respiratory proteins [147]. In contrast to single‐knockout models, mice lacking both PGC‐1α and PGC‐1β suffer lethal cardiac failure soon after birth, attributed to a failure in the perinatal surge of mitochondrial biogenesis [148]. This suggests that both coactivators play essential and complementary roles in the development and maturation of cardiac mitochondrial function during the perinatal period. Their complementary functions extend to skeletal muscle, where enhanced PGC‐1α expression enhances the development of slow, oxidative type I and IIA muscle fibres [149]. In contrast, overexpression of PGC‐1β in skeletal muscle specifically drives the development of type IIX fibres, which are characterised by high oxidative capacity and fast‐twitch properties [150]. This shift is linked to the elevation of OXPHOS and FAO genes, leading to increased mitochondrial mass. Therefore, these two factors can enact different differentiation pathways in muscle fibre types. Of note, PGC‐1α and PGC‐1β display opposing circadian regulation; PGC‐1α levels rise at night and during fasting, whereas PGC‐1β is stimulated by dietary fatty acids and exhibits a diurnal rhythm [151, 152]. In line with this, PGC‐1β‐null mice exhibit significantly lower activity during the night, despite the fact that mice are nocturnal and typically engage in feeding at that time [153]. Furthermore, PGC‐1α has been demonstrated to modulate core clock genes, linking metabolic functions with circadian rhythms [154]. And PGC‐1β is also negatively controlled by acetylation [129], although its post‐translational modifications have been less thoroughly investigated.

In regard to its function in stem cells, there are a few reports. For example, during myogenesis, MyoD engages with the alternative NF‐kB signalling pathway to control PGC‐1β expression. An analysis of ChIP‐seq has demonstrated that MyoD binds specifically to the PGC‐1β gene locus, while it does not associate with PGC‐1α [155]. Moreover, PGC‐1β is essential for proper differentiation of terminal erythroid; alterations in its expression within human HSCs can lead to impaired development of early erythroid progenitors and impaired differentiation, disrupting the cell cycle exit [156]. And intriguingly, in HSCs, telomere dysfunction triggers the activation of p53. This protein then binds to and inhibits the promoters of PGC‐1α and PGC‐1β, establishing a connection between mitochondrial function and telomere integrity [157]. Coincidentally, this telomere–p53–PGC axis also precipitates cardiac aging and hamper myocardial regeneration [158].

#### 2.3.3 PRC

The third member of the PGC‐1 family, PRC, is identified through a database search that revealed similarities in its C‐terminal RNA splicing domain and RNA recognition motif to PGC‐1α [159]. Unlike PGC‐1α and PGC‐1β, which are primarily found in tissues with high energy requirements such as heart, skeletal muscle and brain, PRC is expressed throughout various tissues [113]. Like PGC‐1α and PGC‐1β, PRC engages with nuclear transcription factors related to mitochondrial function, including NRFs, CREB, and ERRα [44, 160, 161]. Silencing PRC in cultured cells results in decreased expression of respiratory chain components and ATP production, accumulation of dysfunctional mitochondria, and inhibition of the G1/S transition in the cell cycle, with PRC levels fluctuating throughout cell cycle progression [159, 162, 163]. When faced with various metabolic stresses, PRC levels significantly increase, promoting the expression of genes associated with proliferation, inflammation and cellular stress [164]. Notably, PRC is involved in regulating pro‐inflammatory genes and proteins associated with DNA damage responses, which help cells exit the cell cycle and offer antioxidant protection, facilitating tissue remodelling [165, 166]. Many of these genes are implicated in inflammatory microenvironments of some cancers and cellular senescence [167, 168].

It is still remains elusive about PRC in development and stem cells. However, it is noteworthy that PRC expression peaks on the first day of embryoid body formation, which parallels its early activation triggered by serum growth factors in cultured cells [159, 161, 169]. Knockout studies in mice have shown that loss of PRC leads to peri‐implantation lethality, highlighting its essential role in the development of early embryo, unlike other family members of PGC‐1 [169]. Moreover, PRC may be involved in facilitating the elevated mitochondrial transcription observed during the early stages of embryogenesis [111]. Additionally, during haematopoietic recovery after 5‐fluorouracil treatment, the expression of PGC‐1s in HSCs varies according to different recovery stages. Specifically, while PRC facilitates stem cell expansion in the early recovery stage, PGC‐1α is crucial for progenitor cells proliferation and the overall recovery of haematopoiesis during later stages [170].

## 3 Retrograde Signalling

**FIGURE 2:**
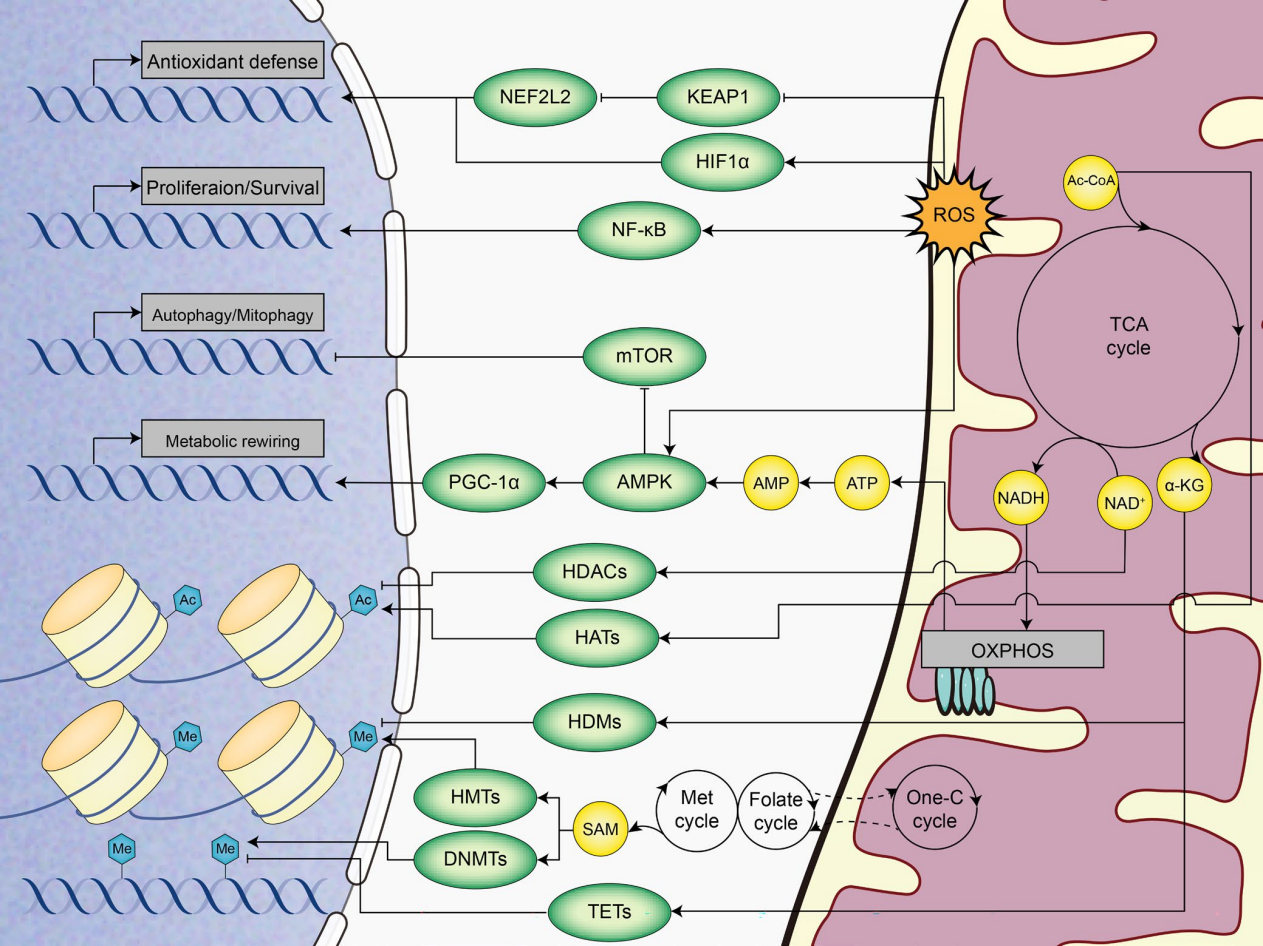
General view of retrograde signalling. Cells induce certain genes transcription through retrograde signalling to cope with various metabolic changes. Mitochondrial dysfunction causes increased ROS, decreased ATP, as well as the accumulation of some metabolites. Elevated levels of ROS prevent KEAP1 from degrading NEF2L2, enabling NEF2L2 to enter the nucleus and trigger the antioxidant defence response. Likewise, HIF1α is stable upon ROS increases and activates certain gene expression. The elevated ROS and the elevated proportion of AMP/ATP also activate PGC1‐α‐mediated mitochondrial metabolism rewiring through AMPK activation. Meanwhile, the activation of AMPK promotes autophagy or mitophagy. Furthermore, metabolites accumulations, such as α‐KG, NAD^+^, Ac‐CoA, alter the epigenetic state of chromatin by affecting key enzymatic activity.

Under certain metabolic conditions, cells initiate particular programs to adapt to ongoing biological changes. To achieve this, they can initiate nuclear target genes transcription through mitochondrial retrograde signals (Figure 2). Mitochondria produce various retrograde signals that help modulate numerous cellular and organismal functions, protecting against dysfunction of mitochondria through promoting nuclear genes expression related to metabolic remodelling and stress response [171]. The retrograde response is present in every organism, though the regulatory mechanisms and pathways may differ [6]. This communication can be activated by changes of energetic challenges, oxidative stress or metabolite levels. Consequently, nuclear epigenetic regulation programs and responses to oxidative and energetic stress are induced, allowing cells to adjust their functions to meet new metabolic demands.

### 3.1 Energetic Stress Responses

Originally identified in yeast, the retrograde signalling or Rtg pathway plays a crucial role in adjusting nitrogen and carbon metabolism by altering nuclear gene expression to mitigate dysfunction of mitochondria [172]. Central to this pathway are the transcription factors Rtg1 and Rtg3, along with the regulatory factor Rtg2, which facilitates the translocation of Rtg1 and Rtg3 into the nucleus in response to fluctuations in ATP levels and mitochondrial membrane potential [173]. These factors stimulate the expression of various metabolic enzymes, activating alternative metabolic pathways such as the glyoxylate cycle to counteract mitochondrial impairment [174]. In organisms beyond yeast, such as *Caenorhabditis elegans* , disruptions in the tricarboxylic acid cycle (TCA cycle) lead to gene expression associated with the glyoxylate cycle, including *gei‐7*, which encodes isocitrate lyase/malate synthase, facilitating energy production independent of mitochondria [175]. On the other hand, in yeast, aside from the activation of Rtg genes, various other pathways can be triggered in response to energy stress. These include Snf1 (which serves a similar role to AMPK in yeast), mTORC1, and sirtuin pathway, working to reprogram metabolism, prevent abnormal histone deacetylation, regulate protein synthesis and improve proteostasis [176, 177, 178].

In mammals, the retrograde response is associated with both AMPK and mammalian target of rapamycin (mTOR); nonetheless, since mammalian cells do not possess a glyoxylate cycle, they employ alternative anaplerotic pathways to adjust their metabolism and address energy shortages. AMPK functions as a sensor of cellular energy, activating when the ratio of AMP to ATP increases. This activation triggers PGC‐1α, which is significant for regulating mitochondrial function [124, 126, 127]. AMPK is also essential for processes like autophagy and mitophagy [179]. Similarly, decreased mTOR activity, often observed during energetic stress, promotes mitochondrial retrograde signalling, while mTOR activation tends to suppress this response [180].

### 3.2 ROS‐Dependent Retrograde Signalling

Mitochondrial dysfunction, such as impaired ETC activity, can lead to the production of harmful by‐products like ROS, which include hydrogen peroxide (H_2_O_2_), superoxide anion (O_2_•^−^) and hydroxyl radicals (OH•), all of which are significant by‐products of aerobic metabolism [181]. Increased ROS generation, often resulting from heightened metabolic activity or mitochondrial impairment, has been connected to the development of multiple diseases, such as diabetes, atherosclerosis, cancer and dementia, as well as the aging process [182]. Up to now, this connection has contributed to the widespread acceptance of the Free Radical Theory of Aging in the field of aging research [183].

While high concentrations of mitochondrial ROS can be harmful to cells, it is now understood that controlled ROS production is essential for modulating redox‐sensitive proteins and triggering various signalling pathways [184]. ROS is crucial for the immune immunomodulatory functions as well as for maintaining overall homeostasis and metabolism [185, 186, 187]. Under normal circumstances, increased levels of ROS in the hypothalamus can suppress appetite, enhance energy expenditure, and promote glucose utilisation in peripheral tissues [188, 189]. Meanwhile, in pancreatic β cells, glucose induces an increase in intracellular H_2_O_2_, which stimulates insulin secretion; conversely, the application of H_2_O_2_ scavengers inhibits this secretion [190]. Intriguingly, plenty of researches indicate that reducing oxidative stress may extend lifespan through a process known as mitohormesis [191, 192, 193]. This concept posits that mild disturbances in mitochondrial function can trigger a cellular response that makes cells less susceptible to future stressors [182]. At the molecular level, ROS produced by mitochondria facilitates these adaptations via various signalling pathways, often by stabilising or regulating the movement of DNA‐binding transcription factors into the nucleus [181]. During redox‐stress conditions, for example, Kelch like ECH associated protein 1 (KEAP1) undergoes oxidation at certain cysteine residues, leading to a structural alteration that inhibits the ubiquitination and degradation of NFE2 like BZIP transcription factor 2 (NEF2L2). This allows NRF2 to accumulate and translocate to the nucleus, where it enhances the transcription of antioxidant and detoxifying enzymes [194]. Additionally, the antioxidant response is enhanced through the stabilisation and nuclear translocation of hypoxia inducible factors (HIFs), as well as the inhibition of prolyl hydroxylases (PHDs) [195, 196, 197]. Elevated mitochondrial ROS levels can also activate nuclear factor kappa B subunit (NF‐κB), which supports cellular proliferation and survival [198].

### 3.3 NAD ^+^‐Dependent Retrograde Signalling

NADH is generated through glycolysis and TCA cycle, whereas nicotinamide adenine dinucleotide (NAD^+^) is restored by NADH oxidation via LDH or ETC complex I [199]. On the other hand, NAD^+^ can be synthesised via two primary pathways: de novo biosynthesis and salvage pathways. The de novo pathway utilises tryptophan and involves a chain of five enzymatic reactions occurring in the cytosol to produce NAD^+^ [200]. Alternatively, the salvage pathway converts precursor molecules like nicotinamide or nicotinamide riboside into NAD^+^ [201]. NAD^+^ levels fluctuate based on metabolic conditions, decreasing with aging and increasing in response to exercise, and various pharmacological or nutraceutical interventions [202]. Higher NAD^+^ levels are associated with improved health span and life span across several model organisms [203]. Both cytosolic and mitochondrial NAD kinase facilitate the conversion of NAD^+^ to NADP^+^, which is crucial for anabolic processes, mitochondrial antioxidant defences and detoxification [204]. Meanwhile, NAD^+^ also serves as a co‐substrate for SIRTs, a family of NAD^+^‐dependent histone deacetylases related to yeast *Sir2* [205, 206]. The deacetylation function of SIRTs is dependent on NAD^+^, linking their function directly to mitochondrial metabolism, as well as the NAD^+^/NADH ratio [202].

SIRT1, SIRT6, and SIRT7 are primarily found in the nucleus and are crucial for deacetylating histones, transcription factors, cofactors and metabolic enzymes. SIRT1, for instance, targets numerous non‐histone proteins that are vital for regulating cellular metabolism, autophagy, mitochondrial biogenesis, cell fate decisions and circadian clock [199, 207, 208, 209, 210, 211, 212]. As the only chromatin‐associated sirtuin, SIRT6 is able to regulate gene transcription [201]. It has been demonstrated to help maintain the levels of blood glucose and support mitochondrial respiration by repressing Hif1α expression [213]. And intriguingly, SIRT6 also modulates the recruitment of CLOCK to gene promoters related to circadian rhythms, particularly those involved in hepatic fatty acid metabolism [214]. SIRT7 involves in maintaining mitochondrial function while its absence leads to metabolic abnormalities [215, 216, 217]. Moreover, SIRT7 is linked to the stem cells regenerative potential, lifespan regulation, age‐related traits and mitochondrial proteostasis [32, 215].

SIRT2 is found mainly in the cytosol. Although SIRT2‐null mice do not exhibit significant metabolic changes, it can target FOXO1 to suppress adipocyte differentiation, ACLY to reduce lipid synthesis and PEPCK1 to stimulate gluconeogenesis [218, 219, 220, 221].

SIRT3, SIRT4 and SIRT5 are mainly localised in mitochondria and SIRT3 seems to be the principal deacetylase among them [222]. SIRT3 influences overall mitochondrial protein acetylation by deacetylating various enzymes, including IDH2, LCAD, HMGCS2, and several components of mitochondrial complex I. This process enhances mitochondrial respiratory chain activity, glucose regulation, lipid metabolism and ketone bodies synthesis [222, 223, 224, 225, 226, 227]. These effects are more pronounced during periods of significant caloric restriction [222]. Other than deacetylation function, SIRT4 also possesses activities related to ADP ribosylation, debiotinylation, and delipoylation [228]. Under nutrient‐rich conditions, it acts as an ADP/ribosyltransferase, preventing glutamine catabolism by targeting glutamate dehydrogenase, thereby limiting the entry of glutamine entry the TCA cycle [229, 230]. In addition, Sirt4‐null mice show disrupted lipid metabolism, which increases exercise tolerance and provides some defence against obesity caused by diet [231]. SIRT5 is crucial for modulating various post‐translational modifications of lysine, such as acetylation, malonylation, glutarylation and succinylation [232, 233, 234, 235]. This protein is known to influence metabolic processes in mitochondria, including amino acid catabolism, TCA cycle activity, and fatty acid metabolism [233]. However, studies have shown that the absence of SIRT5 does not result in significant metabolic disorders, regardless of whether the subjects are on a standard or high‐fat diet [236].

Besides SIRTs, other NAD^+^‐consuming enzymes like PARPs and cADPR synthases also compete for NAD^+^ resources with SIRTs [201]. PARPs assist in transferring ADP‐ribose subunits from NAD^+^ to specific protein targets [237]. On the other hand, cADPR synthases utilise NAD^+^ to generate cADPR, a secondary messenger that plays roles in Ca^2+^ signalling, cell cycle regulation, and insulin signalling [238]. These enzymes contribute not only to metabolic processes, but also to various other functions, including circadian rhythm regulation, DNA repair, signal transduction, inflammation, and cell death [239, 240, 241, 242, 243]. Inhibition of these NAD^+^‐dependent enzymes, whether through pharmacological means or genetic knockout models, results in elevated NAD^+^ levels and subsequent sirtuins activation. This activation is linked to many metabolic improvements observed in animals treated with NAD^+^ precursors [240, 244, 245, 246, 247, 248].

### 3.4 Metabolites and Epigenetic Regulation

Epigenetic modifications influence gene expression without altering the DNA sequence, primarily through dynamic alterations in chromatin structure [249]. The key enzymes in these modifications all rely on mitochondrial metabolic intermediates as cofactors [201]. Histone acetylation is a prominent type of chromatin modification linked to gene activation. Histone acetyltransferases (HATs) add acetyl groups, while histone deacetylases (HDACs) remove them, thereby affecting gene expression by altering chromatin packaging [5]. The cytosolic Ac‐CoA required for histone acetylation is generated by ACLY and ACSS2, which utilise citrate and acetate, respectively [250]. The concentration of Ac‐CoA in cells is influenced by the energy status: high energy production leads to increased Ac‐CoA, enhancing histone acetylation and gene expression, while low energy levels decrease Ac‐CoA, resulting in reduced histone acetylation and suppressed gene expression through chromatin compaction [201]. In contrast, HDACs, including SIRTs, exert demethylase functions that are largely dependent on NAD^+^ levels [202]. Other than acetylation, methylation is another significant epigenetic modification that also relies on mitochondrial metabolites. DNA methylation is catalysed by DNA methyltransferases (DNMTs), while ten‐eleven translocation enzymes (TETs) are responsible for demethylation. Histone methyltransferases (HMTs) and histone demethylases (HDMs) are the enzymes that facilitate the addition and removal of histone methylation, respectively. Both DNMTs and HMTs utilise S‐adenosyl‐methionine (SAM) as the substrate, while demethylases depend on α‐ketoglutarate (α‐KG). Interesting, SAM connects histone methylation to threonine and one‐carbon metabolism [251, 252]. Of note, succinate and fumarate are capable of inhibiting α‐KG‐dependent enzymes, and 2‐hydroxyglutarate (2‐HG), which shares a similar structure with α‐KG, can also inhibit HDMs [253]. Consequently, these modifications heavily depend on the availability of metabolites from the mitochondrial [5].

## 4 Mitonuclear Communication and Proteostasis

**FIGURE 3:**
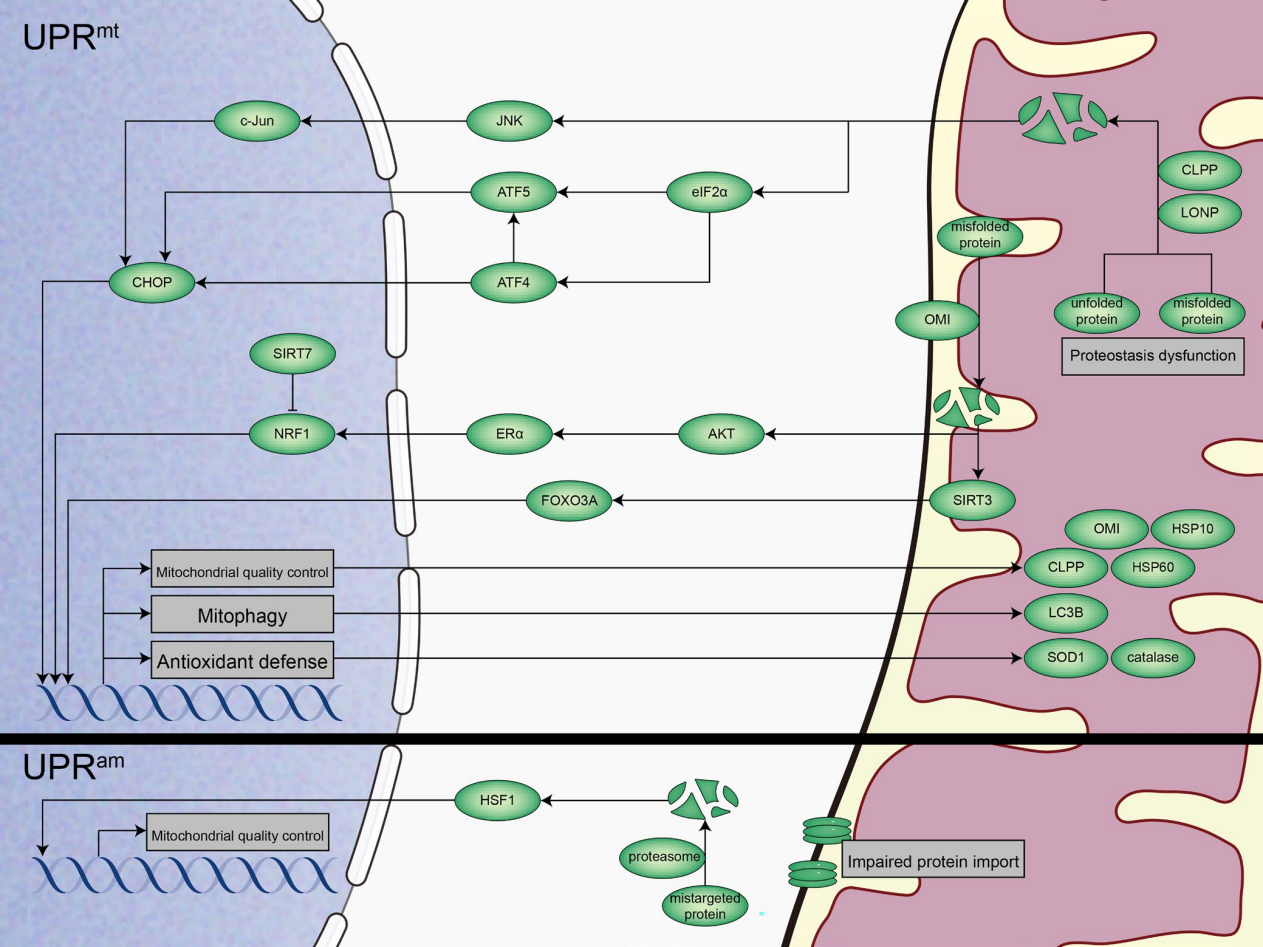
Mitonuclear communication and proteostasis in mammals. Distinct stressors disrupt mitochondrial proteostasis, during this time mitonuclear communication triggers specific transcriptional responses to restore mitochondrial homeostasis. In the UPR^mt^ pathway, the accumulation of unfolded proteins in the mitochondrial matrix activates proteases like CLPP and LONP, which degrade these proteins. The peptides generated after degradation activate JNK signalling via unknown pathways to induce a CHOP‐mediated transcriptional response. Meanwhile, eIF2α is also activated during stress, and the activated eIF2α stimulates ATF4 and ATF5, which in turn induce CHOP. Conversely, when these proteins accumulate in the mitochondrial intermembrane space, OMI degrades mistargeted proteins, triggering transcriptional responses independent of CHOP, including the estrogen receptor alpha (ERα)/NRF1 pathway and SIRT3/FOXO3A pathway. Furthermore, if mitochondrial protein import is disrupted, excess unfolded proteins accumulate in the cytosol, results in stress and activating UPR^am^ pathway. At this point, HSF1 accumulates within the cell and transfers into the nucleus, inducing gene expression that enhance mitochondrial quality control.

Mitochondria possess a protein quality control system primarily composed of proteases and chaperones, which are encoded by nuclear genes. This system is crucial for maintaining mitochondrial proteostasis, as it facilitates the proper folding, assembly, and degradation of mitochondrial proteins under both normal and stress conditions [254]. Different stressors can challenge the quality control systems in the mitochondria. When the quality control system exceeds the load, substantial transcriptional reactions are triggered by mitonuclear communication to restore mitochondrial homeostasis. Two distinct mitonuclear proteostasis responses have been identified: UPR^mt^ and UPR^am^ (Figure 3).

### 4.1 The UPR^mt^

The mitochondrial unfolded protein response (UPR^mt^) is a stress response mechanism governed by specific transcription factors. It is triggered by mitochondrial dysfunction and plays a vital role in regulating gene expression and cellular functions [255]. Mitochondrial dysfunction encompasses various defects, including the accumulation of unfolded proteins and disruptions in OXPHOS due to various stressors like chemical inhibitors and genotoxic stress [256, 257, 258, 259]. Although UPR^mt^ was initially discovered in mammals, its underlying molecular mechanisms have been more extensively explored in *C. elegans* . UPR^mt^ signalling initiates in the mitochondrial matrix of nematodes when misfolded proteins exceed chaperone capacity [260]. The CLPP proteolytic complex digests the excess protein into short peptides ranging from 6 to 30 amino acids. These peptides are subsequently transported from the mitochondrial matrix to the intermembrane space via HAF‐1 and diffuse through the outer mitochondrial membrane into the cytosol [261, 262]. The accumulation of these peptides in the cytosol disrupts mitochondrial import processes through a mechanism that is not well understood, leading to a transcriptional response that is regulated by ATFS‐1 [258]. ATFS‐1 acts as a crucial link between the nucleus and the mitochondria in UPR^mt^ signalling. It has a mitochondrial targeting sequence at the N‐terminal and a nuclear localisation signal at the C‐terminal [6]. Under normal conditions, ATFS‐1 is transported into the mitochondrial matrix and subsequently degraded by LONP. Nevertheless, during mitochondrial stress, the import of ATFS‐1 decreases, enabling it to translocate to the nucleus and activate a protective transcriptional program [258]. In the nucleus, ATFS‐1, along with DVE‐1 and UBL‐5, promotes mitochondrial protein quality control by activating genes encoding molecular chaperones and proteases, like *hsp‐60*, *dnj‐10* and *ymel‐1* [258, 263, 264]. Furthermore, they also enhance the proteins expression that facilitate ROS detoxification, mitochondrial import and protection against mitochondrial dysfunction, ultimately reconstructing mitochondrial homeostasis in vivo [6]. Some glycolytic genes are also induced by ATFS‐1, suggesting that UPR^mt^ can shift cellular metabolism from respiration to glycolysis for ATP production during mitochondrial stress [264].

The UPR^mt^ mechanism in *C. elegans* has been extensively studied. However, due to the complexity and varied mitochondrial functions in mammalian cells, the understanding of the mammalian UPR^mt^ pathway, regulatory mechanisms and regulators are still being explored. Researches on mammalian UPR^mt^ signalling have largely utilised cell culture models, often involving treatments with ethidium bromide or the overexpression of mutant proteins with aggregation tendencies, like ΔOTC, which are directed to the mitochondrial matrix [256, 260]. Some elements of this pathway have been shown to be conserved in mammalian cells. For instance, the accumulation of unfolded proteins in the mitochondrial matrix results in a transient increase in the expression of nuclear‐encoded mitochondrial chaperones such as HSP10, HSP60, mtDNAJ, and the protease CLPP [256, 265]. As mentioned above, CLPP can cleave these unfolded proteins into smaller peptides, which subsequently activate the JNK and CHOP. However, CHOP is also implicated in ER stress responses and is recognised for mediating apoptosis during severe ER stress, raising questions about its specificity in regulating UPR^mt^. Intriguingly, both CHOP and C/EBP have AP‐1 binding sites in their promoters, which are essential for UPR^mt^, but not necessary for gene activation during ER stress [261, 266, 267]. The transcription factor c‐Jun can interact with these AP‐1 site, indicating that the JNK pathway regulates the UPR^mt^ [268, 269]. Another study indicated that ATF5 may be a mammalian direct homologue of ATFS‐1, as it can rescue activation of UPR^mt^ in worms lacking ATFS‐1 [270]. Importantly, ATF5 can respond to mitochondrial stress, with its activity potentially influenced by how efficiently it is imported into mitochondria, much like ATFS‐1. It is crucial for inducing various mitochondrial chaperones and protease genes, supporting its protective role during mitochondrial stress. Activation of ATF5 requires for eIF2 which phosphorylate by PERK and GCN2 [271, 272]. Phosphorylated eIF2 negatively regulate translation of most mRNAs but selectively increase translation of ATF5 and ATF4 which contributes to basal ATF5 transcription [271]. In turn, ATF5 activates the CHOP gene promoter to trigger protective transcriptional program [273]. Of note, some studies suggest that CHOP and ATF4 regulate transcriptional expression of ATF5, implying the existence of cross‐talking [271, 274]. Some researches show that ATF4, ATF5 and CHOP are the potentially ATFS‐1 orthologous transcription factors in mammals, however, it is still a conundrum that these transcription factors function during mitochondrial dysfunction, as the expression of these transcription factors involve integrated stress response and can be triggered under various conditions, like ER stress, viral infection and amino acid depletion [271, 272, 275, 276, 277, 278, 279, 280].

In some cases, a stress response distinct from matrix UPR^mt^ exist specifically sense unfolded proteins located in the mitochondrial intermembrane space, a type of proteolytic stress responses that is independent of CHOP [281, 282]. This response involves a proteolytic process tailored for intermembrane proteins. The process occurs in two primary stages: initially, misfolded proteins targeting the intermembrane space are ubiquitinated and subsequently degraded by the 26S proteasome in the cytosol. After that, any remaining unfolded or excess proteins that accumulate in the intermembrane space are then targeted for elimination by the protease OMI [283]. When there is an overabundance of aggregated proteins in the intermembrane space, it can result in higher levels of ROS and AKT phosphorylation, which in turn activates ERα [281]. ERα then enhances the quality control mechanisms in the intermembrane space by boosting the levels of OMI and NRF1, thus helping to safeguard mitochondrial integrity and maintain mitochondrial potential. Furthermore, the regulatory pathway of UPR^mt^ involves an interaction between SIRT7 and NRF1, which is important for regulating HSCs [32]. Another study has shown that under conditions of stress in the intermembrane space, SIRT3 can be activated independently of CHOP, leading to the deacetylation of FOXO3A. This modification prompts FOXO3A to move into the nucleus, where it triggers the expression of genes involved in ROS detoxification and mitophagy, such as SOD1, catalase, and LC3B [282]. Recently, a study indicates that cells can use the perception of extracellular matrix (ECM) damage to stimulate mitochondrial stress and immune response, thus achieving damage repair and resistance to pathogens [284]. This study reveals that remodelling of ECM, particularly changes in hyaluronan levels, have impacted mitochondrial balance in a manner that is conserved across evolution. Mechanically, ECM remodelling activates the TGF‐β pathway, prompting mitochondrial division and UPR^mt^, ultimately bolstering the organism defence mechanisms against infections. Given the physiological variations across various mammalian tissues affected by mitochondrial dysfunction, it is likely that additional mitonuclear communication pathways exist, activating distinct transcriptional responses.

Some researches have revealed intricate mechanisms by which mitochondrial stress in one tissue can influence distant tissues, ultimately benefiting overall health and lifespan [285]. In *C. elegans* , the germline is pivotal for mediating UPR^mt^ signalling from neuron to intestine. Mitochondria in the germline communicate downstream of neuronal mitokines, Wnt signalling, and serotonin, which in turn regulate lipid metabolic pathways in peripheral tissues and activate UPR^mt^ [286]. When proteostasis is compromised in germline stem cells, it can adversely impact mitochondrial morphology and proteostasis in somatic tissues, triggering the activation of UPR^mt^. A long‐range signalling pathway involving EGL‐20 and Wnt has been identified as key in relaying mitochondrial dysfunction from the germline to somatic tissues [287]. However, male nematodes are less responsive to mitochondrial stress than hermaphrodites [288].

### 4.2 The UPR^am^

Protein import into mitochondria can be hindered by issues such as precursor proteins accumulation that cannot be properly translocated, defective protein translocases, or a decline in membrane potential across the inner mitochondrial membrane. These issues cause precursor proteins to accumulate in cytosol, resulting in proteotoxic stress known as mitochondrial precursor over‐accumulation stress [254]. When mitochondrial protein import is persistently blocked, it triggers significant transcriptional changes, referred to as the unfolded protein response activated by mistargeting of proteins (UPR^am^) [289, 290]. This response helps mitigate the effects of reduced mitochondrial protein import and addresses associated pathologies linked to mitochondrial dysfunction. UPR^am^ involves HSF1, which is bound to molecular chaperones when in non‐stress situations [291].

HSF1 is activated when there is a combination of ROS produced by mitochondria and the accumulation of unfolded mitochondrial precursor proteins in the cytosol because of impaired mitochondrial import. The accumulation of cytosolic ROS leads to the oxidation of cysteine residues in DNAJA1, which releases HSF1 from HSP70. This allows HSF1 to translocate into the nucleus and induce various UPR‐related genes transcription [292, 293]. This process forms functional HSF1 heterotrimers and enhances the expression of genes that contribute to restoring cytosolic proteostasis, including several chaperones, the chromatin‐remodelling factor BRG1, and RPN4, which is crucial for proteasome expression [291, 294, 295]. The increased production of RPN4 subsequently boosts proteasome assembly and activity, aiding in the degradation of non‐imported precursor proteins. Ultimately, UPR^am^ results in reduced expression of various mitochondrial proteins, thereby lessening the protein burden on the translocases [289, 290, 295].

## 5 Mitonuclear Communication in Stem Cells

Stem cells has the unique capability to self‐renew and differentiate into various somatic cell types, and they are found in numerous tissues [296]. These cells are vital for the maintenance and regeneration of tissues throughout the lifespan of multicellular organisms [297]. Recent researches have emphasised the significant role of mitochondria in regulating stem cell activity. In mammals, mitochondria affect the fate and function of stem cells through mechanisms involving metabolic regulation, epigenetic modifications, proteostasis, and mitochondrial dynamics.

### 5.1 Metabolic Regulation

Emerging evidence indicates that the functions of stem cells are heavily influenced by their metabolic states, particularly glycolytic and OXPHOS pathways [298]. While different stem cell types exhibit varying metabolic characteristics, most studies characterise stem cells as predominantly glycolytic [299]. For example, HSCs, MSCs, and ESCs typically contain fewer mitochondria and display an immature mitochondrial structure, aligning with their reliance on glycolysis and limited oxidative respiration [300, 301, 302]. Likewise, hypoxia has been demonstrated to boost self‐renewal and pluripotency in HSCs, MSCs, NSCs, and ESCs [303, 304, 305, 306]. Meanwhile, the shift from oxidative metabolism to glycolysis can promote the reprogramming of somatic cells into induced pluripotent stem cells (iPSCs) [307, 308]. The reliance on anaerobic metabolism might represent an evolutionary adaptation of stem cells in low‐oxygen environments [309]. This can be seen in the low‐oxygen conditions of the bone marrow where HSCs reside, as well as in the CSCs found in solid tumours [310, 311]. By relying on anaerobic metabolism, stem cells can minimise oxidative stress from ROS generated by mitochondria [309]. On the other hand, glycolytic metabolism generates important intermediates that are essential for anabolic processes crucial to stem cells self‐renewal and progenitor cells generation [312]. On the contrary, the energy demands increase during activation and differentiation, necessitating higher levels of ATP and ROS. Indeed, some adult stem cells, including NSCs, HSCs, MSCs and hair follicle stem cells, depend on OXPHOS activity to facilitate differentiation [313, 314, 315, 316, 317]. Thus, glycolysis promotes stemness and inhibits differentiation, as suggested in some studies about ESCs and HSCs [318, 319]. Recent findings also indicate that stem cells exhibit heterogeneity, transitioning between multiple metastable states, which are associated with distinct differentiation potentials and specific transcription factor expression patterns [320]. For instance, a subset of MuSCs with elevated PAX7 levels demonstrates enhanced self‐renewal capabilities and lower metabolic activity compared to a more myogenic subpopulation with lower PAX7 expression. Serial transplantation experiments indicate that only the high PAX7 MuSCs can generate the low PAX7 population, highlighting a hierarchical organisation where elevated ATP levels are associated with commitment to myogenic differentiation, while a quiescent population maintains strong self‐renewal potential [321]. In the differentiation of human trophoblast stem cells into syncytiotrophoblasts, there is a notable reduction in glycolytic flux. These differentiating cells exhibit a sensitivity to glycolytic deficiencies, which can inhibit their differentiation potential and promote inflammation [322]. Furthermore, during alveolar regeneration, the induction of AMPK‐PFKFB2 signalling is critical for upregulating glycolysis, supporting the energy needs for cytoskeletal remodelling in alveolar type II cell differentiation. Aging lung shows diminished AMPK‐PFKFB2 signalling and ATP production in these cells, leading to impaired regeneration capabilities [323].

During their self‐renewal processes, stem cells also rely on mitochondrial FAO alongside glycolysis. As mentioned above, inhibition of FAO or loss of PPARβ/δ can disrupt the asymmetric division of daughter cells in HSCs [87]. However, quiescent adult NSCs exhibit higher levels of FAO compared to their proliferating counterparts and mature neurons [324]. Surprisingly, as MuSCs shift from a quiescent state to active proliferation, they undergo a metabolic change from FAO to glycolysis [325]. And interestingly, in ESCs, HSCs, and NSCs, CPT1, the key enzyme for the FAO pathway, is expressed at higher levels than in their differentiated derivatives [11]. In HSCs, NADPH generated from FAO is critical for cholesterol synthesis, which in turn regulates the biogenesis of extracellular vesicles that influence HSCs fate [326]. Furthermore, research in *Drosophila* shows that haematopoietic progenitors require FAO for differentiation; without it, they exhibit altered histone acetylation, which can be restored by acetate supplementation [327]. More recently, a report shows that circadian rhythm can influence cancer prognosis through the FAO process, with sleep deprivation disrupting the rhythmic patterns of FAO. Mechanistically, dysregulation of the CLOCK protein leads to hyperactivation of ACSL1, which produces palmitoyl‐CoA. This metabolite promotes the S‐palmitoylation of CLOCK at Cys194, establishing a feedback loop that protects CLOCK from ubiquitin‐proteasomal degradation, thereby maintaining cancer stemness despite circadian disruptions [328]. These results indicate that intermediate metabolites derived from FAO are also vital for stem cell fate.

Other evidences highlight the role of ROS as secondary messengers that initiate transcriptional reprogramming [329]. An example of the significance of regulating ROS levels can be seen in uncommitted NSCs. Upon NSCs commitment to progenitor cells during neurogenesis, there is an increase in OXPHOS and ROS levels, which are crucial for triggering the expression of developmental genes [330]. Changes in mitochondria influence stem cell fate by mediating physiological processes driven by ROS, which in turn initiate a dual mechanism that suppresses self‐renew while promoting differentiation through retrograde signalling mediated by NRF2. The FOXO transcription factors family is crucial for regulating antioxidant gene expression, keeping ROS levels low in adult stem cells [331]. NSCs lacking FOXO3 exhibit reduced self‐renewal and diminished capacity to generate various neural lineages [332]. Similarly, HSCs and MuSCs also depend on FOXO3 for sustaining their self‐renewal ability [333, 334]. Interestingly, depriving ESCs of glutamine, a precursor for glutathione synthesis, results in elevated ROS levels. As a consequence, this increase leads to heightened oxidation of OCT4, resulting in reduced OCT4 protein levels and impaired DNA‐binding activity [335]. Nevertheless, excessive ROS can be detrimental, resulting in the exit of quiescence and exhaustion of stem cells, with triggering senescence and apoptosis. For instance, in ATM‐null mice, elevated ROS levels result in HSCs exhaustion via activation of the p38 MAPK pathway, a condition that can be ameliorated by long‐term treatment with antioxidants or p38 MAPK inhibitors [336, 337].

Various types of stem cells demonstrate distinct metabolic needs that depend on the specific cellular context [11]. Notably, these metabolic changes often occur prior to any alterations in transcription and cell fate, indicating that such shifts may act as critical checkpoints for maintaining the integrity and adaptability of cellular functions.

### 5.2 Mitochondria and Epigenetics

Growing evidence suggests a significant cross‐talk between epigenome and mitochondrial metabolism [5]. The presence and availability of mitochondrial metabolites, which also serve as cofactors for epigenetic enzymes, can modify DNA and histones, creating an epigenetic landscape that initiates transcriptional reprogramming in the nucleus [338]. A great number of studies indicate that cellular metabolism directly impacts the epigenome, facilitating a mitonuclear communication that influences chromatin states and cell fate determinations [5, 11].

RegardWit to histone acetylation, it has been widely reported in stem cells. For ESCs, high levels of Ac‐CoA are essential for sustaining enriched histone acetylation, which are crucial for sustaining self‐renewal and pluripotency [339]. Significantly, acetate treatment, a precursor to Ac‐CoA, enhances H3K9/K27 acetylation and postpones early differentiation in a dose‐dependent manner via ACSS2 [339]. And interestingly, the nuclear localisation of ACLY is vital for regulating global histone acetylation levels [340]. Inhibition of ACLY results in reduced histone acetylation in cancer cells and hampers lipid accumulation during adipocyte differentiation, a deficiency that can be reversed by the addition of exogenous acetate through ACSS2 [340]. In addition, overexpression of ACLY boosts MYOD levels and enhances myogenesis through elevated H3K9/K14/K27 acetylation in MuSCs [341]. Collectively, decreased Ac‐CoA levels can drive early differentiation in ESCs by lowering histone acetylation and decreasing chromatin accessibility [339]. In contrast, Ac‐CoA produced by ACLY in adult stem cells is essential for differentiation, partly due to its influence on gene expression through histone acetylation [340, 341]. Of note, other than ACLY, PDC, which transforms pyruvate into Ac‐CoA, is also found in the nucleus of various cell types, where it plays a functional role in histone acetylation. Experiments have shown that isolated nucleus treated with labelled pyruvate produce Ac‐CoA in a dose‐dependent manner, leading to decreased histone acetylation when PDC is silenced [342]. In accord with it, in MuSCs, enhanced PDH activity during proliferation boosts histone acetylation and increases chromatin accessibility at specific genes that need to be silenced for differentiation, thereby promoting self‐renewal [343].

The sirtuin family of HDACs is recognised for its critical function as epigenetic regulators of mitochondrial metabolism, utilising NAD^+^ as a cofactor to deacetylate lysine residues [344]. For example, over 20% of SIRT7‐null mice do not survive beyond their first month, and the remaining knockouts exhibit accelerated aging and significantly reduced lifespans [345]. Mechanistically, SIRT7 is recruited to DNA damage sites, where it deacetylates H3K18, aiding in the recruitment of factors related to the DNA damage response to double‐strand breaks. Moreover, SIRT6 plays a role in epigenetically repressing core pluripotency genes, such as OCT4, SOX2, and NANOG, through targeting H3K56 acetylation and H3K9 acetylation [346]. Furthermore, as MuSCs transition from a quiescent state to proliferation, they undergo a metabolic shift from FAO to glycolysis [325]. This shift lowers intracellular NAD^+^ levels and SIRT1 activity, resulting in higher H4K16 acetylation and stimulation of muscle gene expression. Likewise, in aged mice MuSCs, the reduction of NAD^+^ levels is linked to cellular senescence. Interestingly, increasing NAD^+^ levels through using the dietary supplement nicotinamide riboside, has been shown to mitigate MuSC senescence and extend the lifespan of the mice, with these effects being mediated in part by sirtuin enzymes [248]. Furthermore, sustaining a balance between HATs and HDACs is essential for myogenesis in MuSCs. Evidence suggests that HDAC inhibitors may effectively counteract disease progression by influencing pathogenic gene expression in various muscle‐resident cell types, particularly in individuals with Duchenne muscular dystrophy [347].

Cellular metabolism is vital for supplying essential metabolites that directly affect DNA and histone modifications, while also acting as cofactors and allosteric inhibitors for various epigenetic enzymes. Recent studies have focused on how metabolism impacts chromatin regulation. For example, in ESCs, a lack of threonine results in reduced intracellular SAM, leading to slower stem cell growth and enhanced differentiation [251]. Similarly, methionine deficiency quickly lowers intracellular SAM and trimethylation of H3K4, a characteristic marker of active chromatin, hindering the self‐renewal of iPSCs and increasing their differentiation potential, ultimately resulting in cell apoptosis [348]. However, naive iPSCs maintain low levels of the repressive H3K27 trimethylation as well [349]. The enzyme NNMT, which consumes SAM, is found to be elevated in iPSCs.

When NNMT is knocked down, SAM levels increase, accompanied by H3K27 trimethylation, HIF activation, Wnt signalling repression, and a shift from a naive to a primed state, indicating its role in keeping low levels of SAM and H3K27 trimethylation [349]. On the other hand, naive ESCs maintain a high α‐KG to succinate ratio, leading to DNA and histone hypomethylation that supports a highly accessible genome [350]. Notably, treatment with α‐KG boosts TET‐dependent DNA demethylation and counteracts the rise in H3K27 and H4K20 trimethylation in ESCs, suggesting that the observed trimethylation rises are linked to lower intracellular α‐KG levels [350].

Self‐renewing adult stem cells often reside in hypoxic niches and primarily rely on glycolysis which may result in declined levels of α‐KG. Decreased α‐KG levels cause inhibition of TETs and HDMs to impede DNA and histone demethylation, thereby contributing to sustain the self‐renew ability of adult stem cells. Conversely, activated adult stem cells often display increased OXPHOS, which raises α‐KG levels and promotes histone and DNA demethylation, driving stem cell activation, proliferation, and differentiation [351]. For instance, studies on HSCs reveal that compromised respiration can elevate the 2‐HG/α‐KG ratio, resulting in elevated DNA and histone methylation and a loss of quiescence, since 2‐HG is known to inhibit demethylases [352]. Additionally, metabolites like fumarate and succinate can also competitively inhibit α‐KG‐dependent demethylases, influencing cancer progression [353].

### 5.3 Mitochondrial Proteostasis

The capability to sustain a functional proteome diminishes during the aging process [10]. When mitochondrial proteostasis is abnormal, mitochondrial stress responses are triggered, especially for UPR^mt^. Recently, several studies have indicated that the UPR^mt^ pathway modulates function of stem cells. For instance, adding nicotinamide riboside to the diet of mice has been found to boost NAD^+^ levels, enhancing mitochondrial function partly through the activity of SIRT1. This supplementation also enhances the expression of genes involve in UPR^mt^ and prohibin proteins, which helps prevent senescence in various stem cells, including muscle, neural, intestinal, and melanocyte stem cells in aging mice, thereby supporting longevity [248, 354]. In tune with it, increased NAD^+^ levels resulting from overexpressing NAMPT can attenuate cell senescence in aged MSCs, as NAMPT is the rate‐limiting enzyme in NAD^+^ salvagee [355]. On the other hand, NAD^+^ metabolism is crucial for the anti‐inflammatory functions of MSCs during inflammatory responses [356]. UPR^mt^ also has an important function in HSCs maintenance [282, 357, 358]. As mentioned above, SIRT3 regulates an arm of the UPR^mt^ via deacetylates FOXO3A [282]. However, SIRT3 levels decline with age, and enhancing SIRT3 expression in aged HSCs can improve their regenerative abilities [357]. Meanwhile, FOXO3A deficiency in HSCs shows increased phosphorylation of p38 MAPK, an elevation of ROS, defective maintenance of quiescence, and impaired haematopoiesis [358]. Except for SIRT1 and SIRT3, SIRT7 also has been identified as another regulator of the UPR^mt^ [32]. Similar to SIRT3, SIRT7 expression decreases in aged HSCs, and increasing its levels can improve the regenerative capacity of these cells. The UPR^mt^ can induce SIRT7 expression, which in turn represses NRF1 activity. The relationship between SIRT7 and NRF1 is crucial for reducing mitochondrial translation, thereby helping to restore homeostasis while also repressing energy metabolism and cell proliferation [32]. Accordingly, certain levels of UPR^mt^ signalling may help preserve functions of stem cells during aging, likely through inhibiting senescence.

However, UPR^mt^ is not always beneficial for stem cell function. For instance, studies have shown that increased expression of epithelial‐specific CHOP can lead to cell cycle arrest in mice, which negatively affects wound healing and diminishes intestinal epithelial cells proliferation during inflammatory responses and injuries [359]. Moreover, tissue‐specific loss of HSP60 triggers UPR^mt^, resulting in the loss of stemness in ISCs and hindering the proliferation of the intestinal epithelium [360]. Furthermore, the UPR^mt^ is believed to enhance cancer cells survival, thereby facilitating the growth of tumour [361, 362]. It is also considered a potential therapeutic target in CSC therapy. It may also be potential therapeutic targets for CSCs therapy [363]. Although lots of progress has been made, the role of UPR^mt^ is intricate and further researches are necessary to clarify the implications of mitochondrial proteostasis in stem cell biology.

### 5.4 Mitochondrial Dynamics

Mitochondria of stem cells undergo dynamic processes, including mitophagy, fusion and fission. Recently, the dynamics of mitochondria have been associated with stem cell function and the aging [309]. Unlike post‐mitotic cells, stem cells exhibit fragmented mitochondrial networks, indicating increased fission and decreased fusion [309]. For example, ESCs possess a limited number of immature, globular mitochondria clustered around the nucleus, featuring poorly developed cristae. As these cells undergo differentiation, their mitochondria become larger and more complex, developing more mature cristae and enhancing ATP production alongside ROS generation [364]. Moreover, inhibition of mitochondrial fission GTPase DRP1 prevents iPSC reprogramming, consistent with its significant role in ESC pluripotency [365, 366]. In tune with it, mitophagy is essential for robust iPSC reprogramming; the absence of PINK1, which facilitates mitochondrial clearance via mitophagy, disrupts this process [367]. The limited mitochondrial presence and immature mitochondrial structures in ESCs may protect against detrimental mitochondrial changes, helping to maintain their stemness. A reduction in DRP1 activity in ESCs leads to decreased fission, triggering apoptosis during embryonic development, resulting in the elimination of approximately 35% of embryonic cells in mice before gastrulation [368].

Indeed, emerging evidence highlight the essential role of mitochondrial dynamics in sustaining stem cell homeostasis. For example, different patterns of mitochondrial morphology have been observed during the osteogenic and adipogenic differentiation of MSCs, with fission occurring in late stage of osteogenesis and fusion predominant in adipogenesis [369]. In the in vitro differentiation of ESCs into cardiomyocytes, significant mitochondrial elongation is noted, accompanied by increased levels of MFN2 and OPA1, which regulate outer and inner mitochondrial membrane fusion, respectively [370]. Disruption of either MFN2 or OPA1 impairs the differentiation of ESCs into beating cardiomyocytes, but this can be reversed by restoring their expression [370]. Similarly, MFN2 deficiency impacts the self‐renewal and repopulation abilities of lymphoid‐biased HSCs, while showing little effects on HSCs biased toward myeloid differentiation [371]. Furthermore, in NSCs, alterations in mitochondrial dynamics influence ROS signalling, which in turn regulates self‐renewal and differentiation through NRF2‐mediated mitonuclear communication [330]. Of interest, regions rich in NSCs in the mouse dentate gyrus show higher mitophagy rates [372]. And reportedly, NSCs display an elongated mitochondria morphology in vivo [330]. As NSCs differentiate, their mitochondria shift from an elongated to a fragmented state, before returning to elongated forms in mature postmitotic neurons [330]. During the critical period of neuronal development, a brief rise in mitochondrial fusion helps stabilise these elongated structures in dendrites, supporting synaptic plasticity [373]. Moreover, in aged MuSCs, failure of autophagy results in senescence characterised by breakdown of proteostasis, dysfunction of mitochondria, and elevated oxidative stress, ultimately reducing MuSC function and numbers [374]. However, this senescence can be reversed by restoring autophagy [374]. Besides, through activating fission or preventing fusion, the re‐establishment of mitochondrial dynamics can restore regenerative functions in fission‐impaired or aged MuSCs [375].

SIRT1, FOXO, and mTORC1 pathways are associated with the regulation of autophagy in stem cell activation, reprogramming, and survival under stress, but their specific roles in mitophagy remain to be established [13]. While it is known that metabolism and mitochondrial dynamics affect each other, the direct alterations in mitochondrial dynamics can initiate metabolic reprogramming, which facilitates flexibility in determining cell fate [376].

## 6 Conclusions and Perspectives

Recent studies have highlighted the regulatory influence of energy metabolism, mitochondrial dynamics, epigenetics, and proteostasis in the fate determinations of stem cells. However, it is remains elusive to what extent mitochondria affect the stem cell properties and fate. An increasing number of studies focus on the connection between alterations in cell metabolism and the epigenetic regulation of nuclear gene expression, highlighting how metabolites act as cofactors for enzymes that participate in chromatin remodelling [5]. Furthermore, mitochondrial dynamics and mitochondrial stress can control nuclear gene expression through retrograde signalling [6, 309]. Through mitonuclear communication, gene expression patterns are affected, thereby changing the fate determinations and physiological functions in stem cell, particularly during aging.

Despite significant advancements, a large amount questions remain unanswered about how mitochondria maintain stem cell function through their effects on mitochondrial protein quality control, metabolic signalling and epigenetic regulation. Most current research has focused on model organisms, such as *C. elegans* and mice, and validation in human stem cells is still scarce. In the future, it is necessary to further reveal the conservative mechanism of mitochondria in stem cell regulation, especially to verify the role of UPR^mt^ in human stem cell aging and regeneration, so as to promote the transformation of basic research into clinical application. At the same time, the combination of multi‐omics techniques, such as single‐cell RNA sequencing, epigenome and metabolome, with high‐throughput screening techniques will help to systematically resolve the molecular mechanisms of mitonuclear communication during stem cell aging. This will help build network models of stem cell metabolism and fate regulation, providing precise targets for therapeutic design. In addition, activation of UPR^mt^ has shown potential to improve stem cell function and delay aging [248]. Future studies can focus on the following aspects: development of small molecule activators or regulators of the UPR^mt^ pathway; supplementing NAD^+^ precursors, like NR, or enhancing *Nampt* expression through gene editing to stabilise NAD^+^ levels; evaluating the safety and efficacy of UPR^mt^ targeted therapies for multiple age‐related diseases, such as neurodegenerative diseases. By repairing mitochondrial function, activating UPR^mt^ or optimising mitochondrial protein quality control mechanism of stem cells, the potential of stem cells in regenerative medicine may be improved, such as in the treatment of spinal cord injury, myocardial repair and other fields, optimised stem cell strategies are expected to overcome the current technical bottlenecks. On the other hand, because CSCs share many protective mechanisms with normal stem cells [377], it is critical to design therapies that specifically target CSCs. For example, studying the specific functions of chaperone proteins and autophagy mechanisms in CSCs can help find different targets. Gaining insight into these mechanisms can provide therapeutic opportunities for tissue rejuvenation in aging or specific diseases.

In conclusion, mitochondria have a profound influence on the fate determination and function maintenance of stem cells. Delving deeper into UPR^mt^ and mitonuclear communication opens new avenues for improving aging and various diseases. Future research should prioritise human studies, interdisciplinary collaboration and precision medicine to further unravel the multifaceted roles of mitochondria in stem cell biology. By optimising mitochondrial function, we can advance the development of regenerative medicine, enabling precise interventions for tissue rejuvenation and age‐related diseases. Therefore, clarifying the role of mitochondria in physiology and aging of stem cells will be a key scientific challenge in the coming years.

## Author Contributions

B.P. wrote thepaper, H.Z. and Y.W. revised the manuscript. All authors contributed to the article and approved the submitted version.

## Conflicts of Interest

The authors declare no conflicts of interest.

## Data Availability

Data sharing not applicable to this article as no datasets were generated or analysed during the current study.
